# Hydrophobic mismatch sorts SNARE proteins into distinct membrane domains

**DOI:** 10.1038/ncomms6984

**Published:** 2015-01-30

**Authors:** Dragomir Milovanovic, Alf Honigmann, Seiichi Koike, Fabian Göttfert, Gesa Pähler, Meike Junius, Stefan Müllar, Ulf Diederichsen, Andreas Janshoff, Helmut Grubmüller, Herre J. Risselada, Christian Eggeling, Stefan W. Hell, Geert van den Bogaart, Reinhard Jahn

**Affiliations:** 1 Department of Neurobiology, Max Planck Institute for Biophysical Chemistry, D-37077 Göttingen, Germany; 2 Department of NanoBiophotonics, Max Planck Institute for Biophysical Chemistry, D-37077 Göttingen, Germany; 3 Institute for Physical Chemistry, Georg-August-University, Göttingen D-37077, Germany; 4 Institute for Organic and Biomolecular Chemistry, Georg-August-University, Göttingen D-37077, Germany; 5 Department of Theoretical and Computational Biophysics, Max Planck Institute for Biophysical Chemistry, D-37077 Göttingen, Germany; 6 Department of Chemistry, Leibnitz Institute of Surface Modification, D-04318 Leipzig, Germany; 7 MRC Human Immunology Unit, Weatherall Institute of Molecular Medicine, University of Oxford, Oxford OX3 9DS, UK; 8 Department of Tumor Immunology, Radboud University Medical Center, 6525 GA Nijmegen, The Netherlands

## Abstract

The clustering of proteins and lipids in distinct microdomains is emerging as an important principle for the spatial patterning of biological membranes. Such domain formation can be the result of hydrophobic and ionic interactions with membrane lipids as well as of specific protein–protein interactions. Here using plasma membrane-resident SNARE proteins as model, we show that hydrophobic mismatch between the length of transmembrane domains (TMDs) and the thickness of the lipid membrane suffices to induce clustering of proteins. Even when the TMDs differ in length by only a single residue, hydrophobic mismatch can segregate structurally closely homologous membrane proteins in distinct membrane domains. Domain formation is further fine-tuned by interactions with polyanionic phosphoinositides and homo and heterotypic protein interactions. Our findings demonstrate that hydrophobic mismatch contributes to the structural organization of membranes.

## Abstract (web summary)

Clustering of proteins in the plasma membrane plays an important role in the regulation of both cellular signalling and membrane remodelling. Milovanovic *et al.* demonstrate that mismatch between transmembrane domain length and the lipid bilayer thickness is sufficient to drive clustering of SNARE proteins.

## Graphical abstract

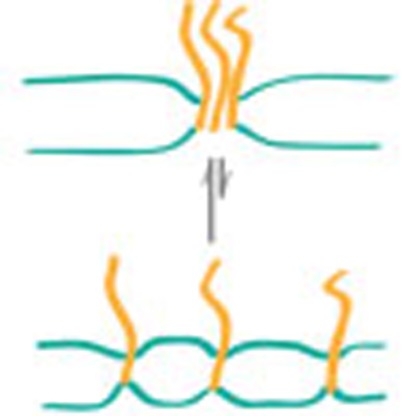

Since many years, the architecture of biological membranes has been subject of intense research. Presently, the concepts for the arrangement of membrane proteins, particularly in the plasma membrane of eukaryotic cells, are undergoing major changes. According to the classical Singer–Nicolson model membrane proteins are viewed as separate entities floating in the lipid bilayer like icebergs in a sea. In contrast, it is now appreciated that many (if not all) membrane proteins are not evenly distributed across the membrane but rather organized in microdomains with a high local protein concentration, ranging in size between 2 and 200 nm (refs 1, 2, 3, 4). Frequently, domain formation appears to be essential for the functions governed by these proteins, for example, by forming localized hotspots for signalling or for exo and endocytosis^5^,^6^,^7^.

SNARE proteins operating in exocytosis at the plasma membrane, in particular syntaxins 1 and 4, have served as convenient models for studying mechanisms responsible for protein clustering^8^,^9^,^10^. Syntaxins are tail-anchored membrane proteins mostly with a single hydrophobic transmembrane domain (TMD) at the C terminus^11^. In the plasma membrane, these proteins are organized in clusters with a diameter of ~70 nm that are dependent on the presence of cholesterol in the membrane, but are distinct from clusters formed by ‘classical raft’ residents such as caveolin or proteins linked to a glycosylphosphatidylinositol anchor^5^,^12^,^13^. In addition, clustering is dependent on the presence of plasma membrane-specific polyphospohoinositides (PI(4,5)P_2_ and PI(3,4,5)P_3_) that bind to a conserved juxtamembrane polybasic motif^6^,^10^,^14^,^15^,^16^,^17^,^18^. However, the physical principles underlying cluster formation within the bilayer are still unclear, with explanations including mechanisms as diverse as phase partitioning into cholesterol-enriched membrane rafts^5^,^19^,^20^,^21^, decreased solubility caused by the presence of cholesterol^15^,^16^, electrostatic interactions with the phosphoinositides^10^,^18^, and homophilic as well as heterophilic interactions between the proteins themselves involving either the hydrophobic TMDs or the hydrophilic domains^8^,^9^,^22^. In any case it is becoming apparent that membrane cholesterol plays a fundamental role in domain formation that is fine-tuned by the other factors but cannot be replaced by them. The causative mechanism of cholesterol is still unclear, with neither phase partitioning (yielding phase-segregated areas of much bigger size which exclude many of the cholesterol-dependent proteins^4^,^23^) nor specific binding of cholesterol to individual proteins (shown for only few of the cholesterol-dependent proteins) providing a satisfying explanation. A second important point is that closely homologous SNARE proteins such as syntaxin 1 and 4 are segregated into non-overlapping membrane domains^8^. Understanding how syntaxins segregate into distinct membrane clusters is essential for understanding their distinct roles in regulated (syntaxin 1) and constitutive (syntaxin 4) exocytosis.

In this study, we have investigated whether hydrophobic mismatch may be the underlying physical principle for many of the effects summarized above, with the effects of cholesterol being due to effects on membrane thickness. Hydrophobic mismatch means that the length of the hydrophobic part of a membrane-spanning macromolecule does not match the thickness of the hydrophobic core of the membrane. Such mismatch results in hydrophobic ‘defects’ at the boundary between protein and membrane lipid, which impose an energy penalty that can be minimized by clustering (Fig. 1a). The plasma membrane is a complex milieu with diverse thicknesses, amendable to quick changes, and we reasoned that the patterning of the plasma membrane might be caused by sequestering of lipids and proteins in membrane regions of matching hydrophobic thickness. Indeed, mismatch-driven clustering was previously shown to occur in a system employing synthetic peptides and artificial membranes^24^,^25^. Furthermore, hydrophobic mismatch was invoked as possible mechanism for the retention of membrane proteins in early compartments of the secretory pathway (such as the endoplasmic reticulum (ER)) since membrane thickness increases between the ER and the plasma membrane^26^. However, there appear to be many exceptions^27^,^28^, and thus hydrophobic mismatch is presently not being considered as a relevant factor contributing to membrane patterning and protein sorting. In this study, we show that hydrophobic mismatch between the length of TMDs and the thickness of the plasma membrane contributes to the clustering of proteins in the plasma membrane. We also show that hydrophobic mismatch can contribute to the segregation of structurally closely homologous SNARE proteins in distinct membrane domains even when the TMDs differ in length by only a single residue.

## Results

### Clustering of syntaxin isoforms by hydrophobic mismatch

**Figure 1:**
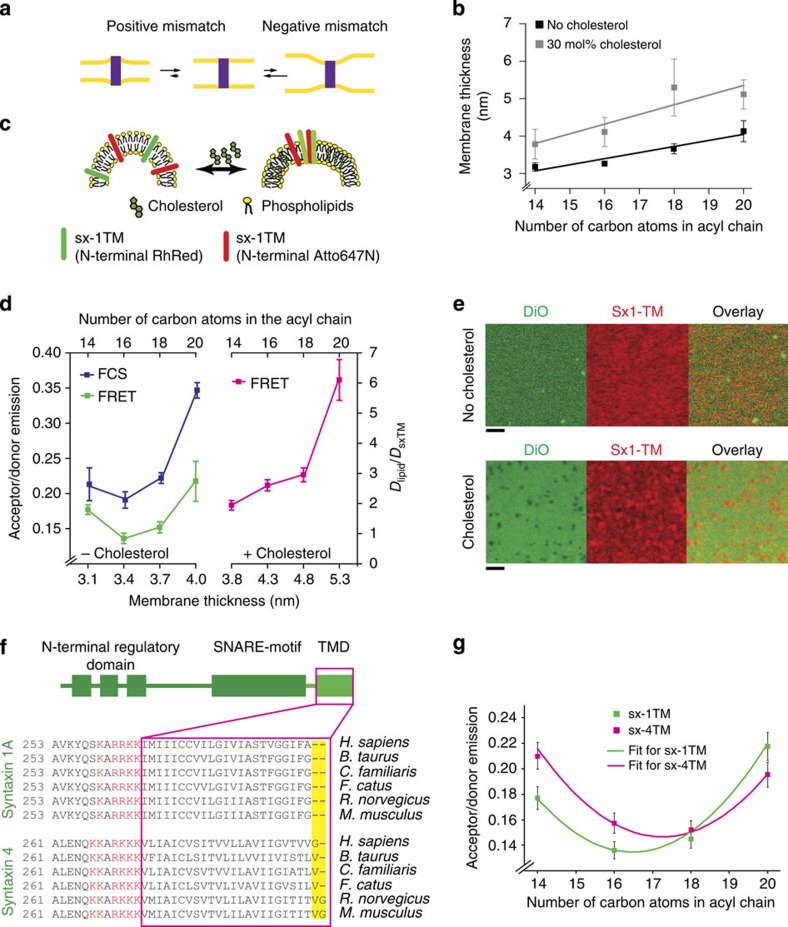
Clustering of syntaxin isoforms by hydrophobic mismatch. (**a**) Positive and negative hydrophobic mismatch caused by differences between membrane thickness and TMD length. (**b**) Bilayer thickness determined by imaging ellipsometry of supported lipid bilayers composed of C14:1, C16:1, C18:1 and C20:1 PC with and without 30 mol% cholesterol (three independent experiments±s.d.). Solid lines show linear regression analyses (slopes of 0.15 and 0.25 for without and with cholesterol, respectively). (**c**) Scheme of the clustering assay for sx-1TM in 100 nm sized liposomes using FRET. A mixture of sx-1TM was used that was N-terminally labelled with Rhodamine Red and Atto647N. (**d**) Clustering determined by FRET using liposomes composed of PC of increasing acyl-chain lengths, without (green) and with (pink) 30 mol% cholesterol. Independently, normalized lateral diffusion coefficients of sx-1TM labelled with Atto647N were determined by FCS (blue). Error bars: range from two independent reconstitutions, three technical repeats each. (**e**) Clustering of sx-1TM (labelled with Rhodamine Red) in supported lipid bilayers (C18:1 PC) in the absence (top) and presence (bottom) of 30 mol% cholesterol measured by confocal microscopy. The membrane was visualized with the lipophilic dye DiO. (Scale bars, 2 μm) (**f**) Domain organization and alignment of the TMD regions of syntaxin 1 and syntaxin 4. The TMDs and adjacent polybasic patches are marked in magenta and red, respectively. (**g**) Clustering of human sx-1TM (green) and sx-4TM (magenta) measured by FRET without cholesterol as in **d**. Solid lines show fits with quadratic curves. Error bars: range from two independent reconstitutions, three technical repeats each.

To dissect the protein distribution in different membrane environments, we started by addressing syntaxin clustering in artificial liposomes with defined membrane thicknesses. First, we determined the effect of acyl-chain length and cholesterol on membrane thickness. To this end, we prepared ~100 nm sized large unilamellar vesicles (LUVs) composed of unsaturated phosphatidylcholine (PC) with stepwise increase in the length of the acyl chains (ranging from C14:1 to C20:1) either in the absence or in the presence of 30 mol% cholesterol. The thickness of these membranes was determined by imaging ellipsometry measurements (Fig. 1b), which is based on polarization changes of monochromatic light upon reflection on the bilayer^29^. As expected^30^, the membrane thickness increased by about 0.15 nm for each carbon unit added to the phospholipid acyl chains. Inclusion of 30 mol% cholesterol increased the membrane thickness by ~0.8 nm, independently of the acyl-chain length (Fig. 1b), indicating that cholesterol is a main modulator of membrane thickness.

We choose the SNAREs syntaxin 1 and syntaxin 4 to study the influence of hydrophobic mismatch on the distribution of membrane proteins for two reasons. First, the lengths of the hydrophobic segments of these SNAREs (21–23 amino acids) appear to be shorter than that needed to fully span the average hydrophobic core of a plasma membrane. This is particularly evident from the crystal structure of the neuronal SNARE complex where the TMD segments of the SNAREs synaptobrevin 2 and syntaxin 1 seem indeed not sufficiently long to traverse the average thickness of plasma membrane of eukaryotic cells (~4 nm). In a simulation, this resulted in defects in lipid packing^30^,^31^ and suggested that these SNAREs might prefer, or even organize, membrane regions of lipids with matching thicknesses. Second, these two syntaxins segregate in separate clusters although they are homologous and structurally very similar to each other. While it was shown previously that segregation depends at least in part on homophilic interactions between the cytoplasmic domains^22^, it is conceivable that the small differences in the length of the TMD segments (see below) may contribute to segregation. To isolate the effects on clustering within the membrane space from ‘secondary’ effects caused by protein–protein interactions in the hydrophilic space, we employed truncation mutants of syntaxins 1 and 4 with their cytoplasmically oriented domains deleted.

We tested whether syntaxin clustering is dependent on membrane thickness. For this purpose we measured clustering of the syntaxin 1 truncation mutant (sx-1TM) by Förster resonance energy transfer (FRET) using an approach very similar to that described by Murray and Tamm^15^,^16^. Two sx-1TM populations labelled with spectrally separated fluorophores were mixed and incorporated into the liposomes described above, resulting in a high FRET signal in case of cluster formation (Fig. 1c). We found that in the absence of cholesterol, the FRET efficiency of sx-1TM was lowest (that is, least protein clustering) in membranes composed of C16:1 PC compared with C14:1, C18:1 and C20:1 PC (Fig. 1d). In the presence of cholesterol, clustering of syntaxin 1 was strongly enhanced (about 50% increase in FRET efficiency, Fig. 1d) for a given acyl-chain length, but was similar when related to membranes with the same thickness without cholesterol. As an independent approach we also determined the lateral mobility of sx-1TM by fluorescence correlation spectroscopy (FCS) in stacked supported lipid bilayers. Lateral mobility is expected to inversely correlate with cluster formation. As shown in Fig. 1d, a profile very similar to the FRET measurements was obtained, with sx-1TM showing the highest mobility in membranes of C16:1 PC. Together, our data show that the syntaxin 1 clustering was lowest in membranes composed of C16:1 PC, corresponding to a hydrophobic thickness of about 3.4 nm, well below the average thickness of a eukaryotic plasma membrane. Clustering increased with increasing membrane thickness independent of whether this increase was caused by longer acyl chains or by the inclusion of cholesterol in the membrane.

To directly visualize syntaxin clustering in dependence of cholesterol, we prepared supported artificial bilayers (C18:1 PC, with or without 30 mol% cholesterol) containing sx-1TM. In this experiment sx-1TM was labelled with the dye Atto647N to monitor its distribution, with the membrane being stained with the green fluorescent lipid analogue DiO (3,3′-dilinoleyloxacarbocyanine; Fig. 1e). Clustering was clearly observable in the cholesterol-containing membranes, whereas it was much less conspicuous in the absence of cholesterol, in agreement with the results described above. Clearly, clustering is not due to cholesterol-induced phase separation of membrane lipids since these membranes did not contain any lipids with saturated fatty acids required for partitioning into Lo and Ld phases. Rather, our experiments reveal that the effect of cholesterol is caused by the increase of the membrane thickness, which results in clustering due to increased hydrophobic mismatch.

Interestingly, the TMD of syntaxin 4 is longer by 1–2 amino acids than that of syntaxin 1, and this difference in length is well conserved in mammalian species (Fig. 1f). Since the length of the TMD determines the hydrophobic matching with the surrounding lipid environment^24^,^25^, it is expected that optimal matching (that is, lowest clustering) requires a thicker bilayer for syntaxin 4 than syntaxin 1. Therefore, we compared clustering of syntaxin 1 with that of syntaxin 4 as a function of membrane thickness, using an analogous truncation mutant for syntaxin 4 that lacked most of the cytoplasmic part (sx-4TM). Indeed, the local minimum of sx-4TM clustering was observed in C18:1 PC membranes instead of C16:1 PC membranes observed for sx-1TM (Fig. 1g). These FRET data fit well with a quadratic curve (*ax*^2^+*bx*+*c*). In this empirical model, −*b*/(2*a*) reflects the acyl-chain lengths with the lowest clustering which are 16.5 and 17.3 for sx-1TM and sx-4TM, respectively. Based on our imaging ellipsometry (Fig. 1b), these acyl-chain lengths correspond to membrane thicknesses of 3.6 nm for sx-1TM and 3.7 nm for sx-4TM. Together, our data indicate that the clustering of TMDs is indeed determined by the hydrophobic matching with the local lipid environment; with a length difference of even a single residue resulting in a shift towards an ~1-Å thicker membrane area. Despite this difference, both syntaxins are expected to cluster since the plasma membrane has an average thickness of around 4 nm (ref. 30), which would result in pronounced sequestering of these proteins to regions of decreased thickness.

### Synergy of ionic interactions and hydrophobic mismatch

**Figure 2:**
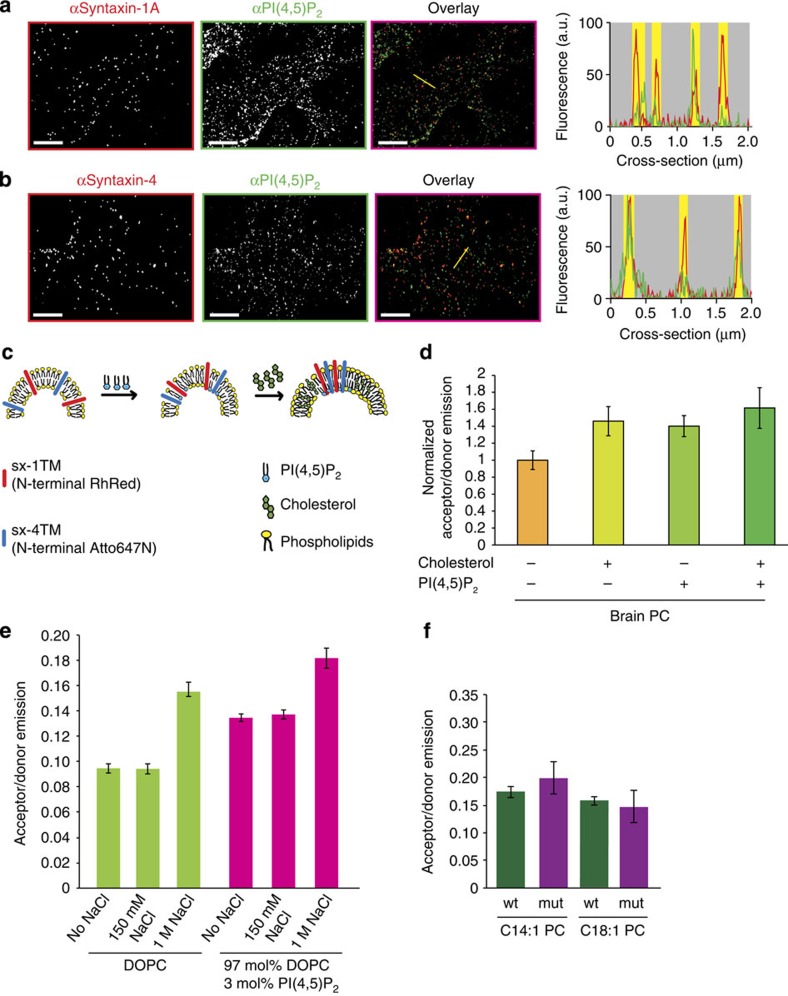
Ionic interactions and hydrophobic mismatch act synergistically on syntaxin clustering. (**a**,**b**) Both syntaxin 1 and syntaxin 4 clusters colocalize with PI(4,5)P_2_. Plasma membrane sheets derived from PC12 cells were immunostained for PI(4,5)P_2_ (green), syntaxin 1 (**a**; red) and syntaxin 4 (**b**; red) and imaged by two-colour STED microscopy. The graphs show the fluorescence intensity profiles as indicated in the figures (syntaxin 1 and 4: red profiles; PI(4,5)P_2_: green profiles); yellow bars depict the positions of the domains. (**c**,**d**) Both PI(4,5)P_2_ and cholesterol enhance co-clustering of sx-1TM and sx-4TM. FRET was measured after reconstitution in large unilamellar vesicles containing sx-1TM labelled with Rhodamine Red (donor fluorophore) and sx-4TM with Atto647N (acceptor) composed of porcine brain PC without or with 1 mol% PI(4,5)P_2_ and/or 30 mol% cholesterol (± range from two independent reconstitutions, three technical repeats each). (**e**) Clustering of sx-1TM by FRET in the presence or absence of 150 mM or 1 M NaCl and in DOPC liposomes in the absence or presence of 3 mol% PI(4,5)P_2_. (**f**) Clustering of sx-1TM (green) and the sx-1TM oligomerization mutant (purple), monitored by FRET, in C14:1 PC or C18:1 PC liposomes. Error bars: range from two independent reconstitutions, three technical repeats each. Scale bars, 2 μm.

Next we investigated how clustering caused by hydrophobic mismatch is influenced by phosphoinositides. Syntaxin 1 is known to interact with PI(4,5)P_2_ and/or PI(3,4,5)P_3_ via a conserved polybasic motif directly adjacent to the hydrophobic TMD (Fig. 1f)^6^,^10^,^15^,^16^. Furthermore, both phosphoinositides are highly accumulated in at least a fraction of syntaxin 1 clusters in the plasma membrane^6^,^10^,^17^,^18^. Using two-colour super-resolution stimulated emission depletion (STED) microscopy, we confirmed the enrichment of PI(4,5)P_2_ in syntaxin 1 clusters within plasma membrane sheets prepared from neuroendocrine PC12 cells (Fig. 2a). Syntaxin 4 also contains a polybasic motif at the membrane interface and, indeed, PI(4,5)P_2_ was also enriched in syntaxin 4 clusters (Fig. 2b). In the plasma membrane, the density of PI(4,5)P_2_ clusters (13.9±1.6 clusters per μm^2^) was approximately three times higher than the cluster density of syntaxin 1 (4.5±0.4 clusters per μm^2^) and syntaxin 4 (5.4±0.7 clusters per μm^2^), which is not surprising when considering that PI(4,5)P_2_ interacts with many other proteins in cells^32^,^33^. We then reconstituted sx-1TM labelled with Rhodamine Red (donor fluorophore) and sx-4TM labelled with Atto647N (acceptor fluorophore) in LUVs and measured interaction by FRET (Fig. 2c). The presence of 1 mol% PI(4,5)P_2_ in LUVs composed of brain PC caused an increase of the FRET efficiency, indicating that the TMDs of the two syntaxin isoforms co-clustered in the membrane when PI(4,5)P_2_ was present. This clustering corroborates with our previous findings showing that interactions with polyanionic phosphoinositides can cluster syntaxin 1 refs 10, 18. Clustering was also observed in the presence of both PI(4,5)P_2_ and cholesterol (Fig. 2d) showing that interactions with phosphoinositides act synergistically with hydrophobic mismatch. To further dissect how electrostatic repulsion between the polybasic linker region affects clustering by hydrophobic mismatch, we repeated our FRET assay in the presence of high concentrations of NaCl. Membrane clustering of sx-1TM was strongly promoted when electrostatic interactions were screened under the presence of 1 M NaCl. This indicates that the repulsion of the polybasic linkers of syntaxins counteracts hydrophobic mismatch and thereby limits clustering even in the presence of PI(4,5)P_2_ (Fig. 2e).

For a better understanding of the membrane clustering, we carried out coarse-grain molecular simulations. In these simulations, clustering of sx-1TM in membranes composed of longer (C20:1) and shorter (C12:1) acyl chains was observed already at very short simulation time scales (~100 μs). The simulation qualitatively reproduced the experimentally observed minimum of sx-1TM clustering in C16:1 membranes (Supplementary Fig. 1). However, a detailed analysis revealed that hydrophobic mismatch is not the only mechanism driving clustering. In fact the mismatch between the sx-1TM peptide and the membrane seems to be always negative even in thin membranes (Supplementary Fig. 2a,b). In such thinner membranes, syntaxin clustering was primarily promoted by increased homotypic protein–protein interactions, which for membranes with shorter acyl chains were facilitated by the much larger rotational tilting angle relative to sx-1TM tilting angles in thicker membranes (Supplementary Figs 1b and 2c,d)^34^. In membranes with longer acyl chains, protein clustering was mainly caused by the more pronounced hydrophobic mismatch (Supplementary Fig. 2a), which causes a corresponding free energy penalty ΔΔ*G* (Supplementary Fig. 2b), proportional to the protein–lipid mismatch interface. While strictly a free energy difference, the effective TMD extension (*d*) of the mismatch interface is rather constant for a given membrane and, therefore, the length of the protein–lipid interface mainly determines this free energy. Accordingly, and intuitively, we will refer to ΔΔ*G*/*d* as a ‘line tension’.

We then further characterized the influence of homotypic TMD interactions on SNARE clustering. It was demonstrated previously that syntaxin 1 TMDs homodimerize in membranes despite the electrostatic repulsion of the cationic linker. This homodimerization depends on specific protein–protein interactions in the hydrophobic phase that can be disrupted by alanine substitutions of three hydrophobic side chains within the TMD (M267A, C271A and I279A)^35^. To examine whether homodimerization contributes to homophilic clustering during hydrophobic mismatch, we reconstituted the corresponding sx-1TM mutant peptides and measured clustering by FRET. In membranes composed of C14:1 PC and C18:1 lipids (that is, both thinner and thicker than required for optimal hydrophobic matching of syntaxin 1), the dimerization mutant clustered comparable to wild type (Fig. 2f), indicating that at these conditions homotypic interactions were not required for clustering. These findings agree with the results from our simulations, where although clustering of the dimerization mutant was reduced compared with wild-type sx-1TM, the pronounced rotational mobility in thin membranes facilitated clustering of even the sx-1TM mutant. Here the larger oligomers were absent and the shape of the cluster distribution resembled the wild-type size distribution in membranes with longer acyl chains (Supplementary Fig. 2c,d). Together, these results demonstrate that the observed minimum of clustering in membranes without cholesterol is caused by a competition between protein–protein interactions and hydrophobic mismatch. For membranes with more pronounced negative mismatch (such as the plasma membrane environment for syntaxins), clustering is mainly driven by the line tension that results from the increased hydrophobic mismatch free energy.

### Segregation of syntaxins 1 and 4 by hydrophobic mismatch

**Figure 3:**
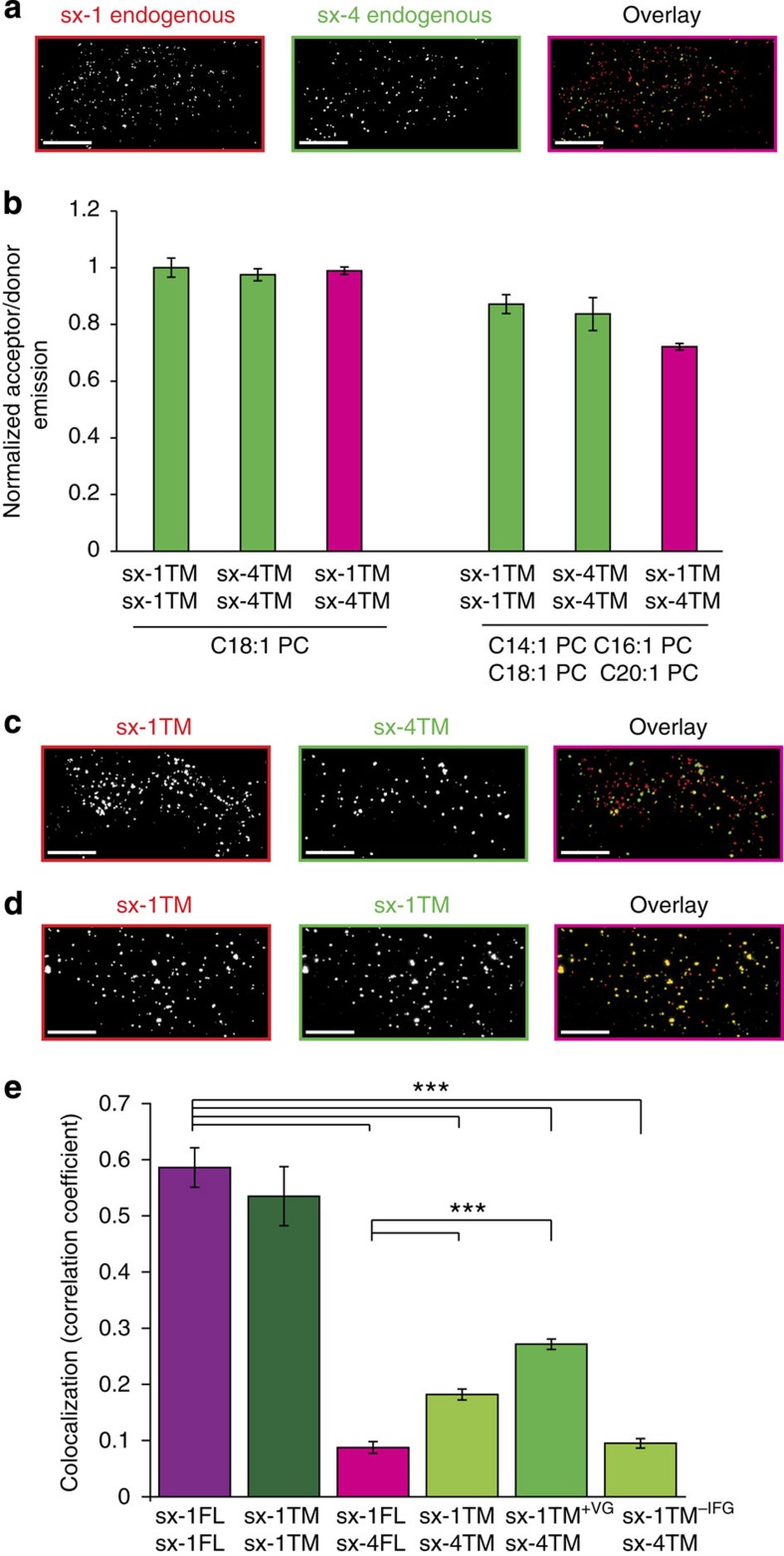
Differences in the length of the TMDs contribute to segregation of syntaxin 1 and 4 to distinct clusters in the plasma membrane. (**a**) Two-colour STED microscopy of PC12 cell sheets immunostained for syntaxin 1 and syntaxin 4 shows segregation of endogenous proteins into separate clusters. (**b**) Reduced co-clustering of sx-1TM and sx-4TM in membranes composed of a mixture of PC with different acyl-chain lengths. FRET assay is similar to Fig. 1c, but now measuring clustering of TMDs in liposomes composed of either C18:1 PC or an equimolar mixture of C14:1, C16:1, C18:1 and C20:1 PC. All liposomes contained 1 mol% PI(4,5)P_2_ and 30 mol% cholesterol (± range from two independent reconstitutions, three technical repeats each). (**c**) Same as in **a** but now using PC12 cell sheets derived from cells expressing truncation mutants of syntaxin 1 and 4 (sx-1TM; sx-4TM) that are fused to mCherry and EGFP, respectively, and immunostained with antibodies against mCherry and EGFP. (**d**) Control experiment of PC12 cells co-expressing sx-1TM tagged with either mCherry or EGFP, showing co-localization (Scale bar, 2 μm). (**e**) Overlap of clusters in membrane sheets from PC12 cells transfected with various sx-1TM and sx-4TM mutants (all mCherry and EGFP tagged, respectively) measured by determining the Pearson-correlation coefficient. Sx-1FL and sx-4FL; full length constructs of syntaxin 1 and syntaxin 4, respectively (from **a**). Each analysis included at least 10 sheets from 3 independent transfections (***P<0.001, two-sided, unpaired *t*-test; error bars show s.e.m.). Scale bars, 2 μm.

In the final set of experiments, we asked whether the small difference in the length of the TMDs between syntaxin 1 and 4 might result in at least partial segregation into separate clusters because of hydrophobic mismatch between the TMDs. Thus, difference in length of the TMDs may contribute, in addition to the well-established homophilic interactions between SNARE motifs^8^,^9^,^22^, to the conspicuous segregation of the two syntaxins into different clusters (Fig. 3a). To address this question, we first reconstituted sx-1TM and sx-4TM in liposomes composed of a mixture of PC with different acyl-chain lengths (C14:1 to C20:1) and measured clustering by FRET. Clustering of sx-1TM with sx-4TM, but not of sx-1TM to sx-1TM and sx-4TM to sx-4TM, was lower compared with liposomes containing only C18:1 PC (Fig. 3b). This indicates that syntaxin TMDs preferentially cluster in regions containing lipids with matching hydrophobic thickness and a heterogeneous plasma membrane environment can drive segregation of membrane proteins with different lengths of TMDs in distinct membrane domains.

We then asked if hydrophobic mismatch can also cause segregation of syntaxin 1 and 4 in the complex environment of a plasma membrane. To this end, we transfected PC12 cells with truncation mutants of both syntaxins (sx-1TM and sx-4TM, similar to the fragments used in abovementioned experiments) N-terminally fused to EGFP and mCherry, respectively. When membrane sheets from these cells were analyzed by two-colour super-resolution STED microscopy^36^, segregation of the two mutants in separate clusters was observed (Fig. 3c–e). To test if this segregation was due to the difference in length of the TMDs, we generated syntaxin 1 TMDs that were either two amino acids longer (sx-1TM^+VG^) or three amino acids shorter (sx-1TM^−IFG^) than wild type. In line with the hydrophobic mismatch hypothesis, clusters of the shorter sx-1TM^−IFG^ strongly segregated from sx4-TM clusters (Fig. 3e). In contrast, the longer sx-1TM^+VG^, with a similar length of TMD as sx-4TM, showed significantly more co-localization with sx-4TM. Thus, our data show that increasing or reducing hydrophobic mismatch by altering the length of TMDs by only a few residues contributes not only to protein clustering but also to segregation into separate clusters.

## Discussion

**Figure 4:**
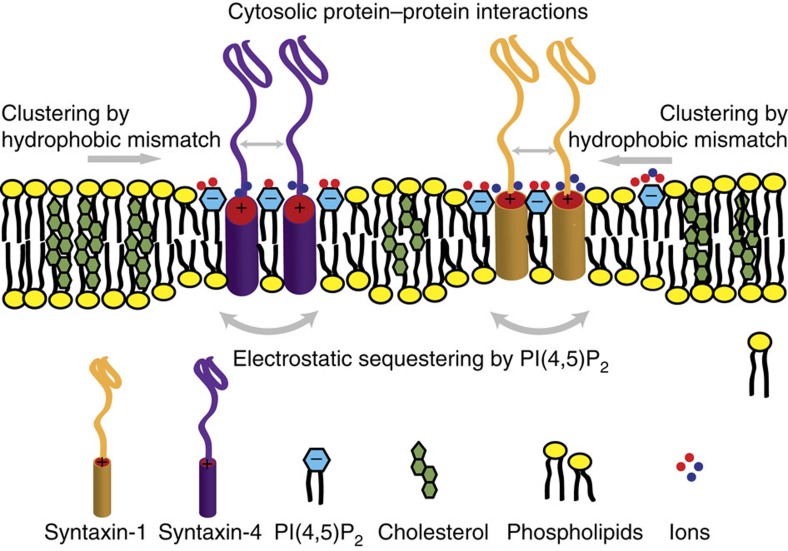
Synergistic model of syntaxin clustering in the membrane. Syntaxin membrane clustering is induced by a combination of hydrophobic mismatch (increased by cholesterol-induced membrane thickening) and electrostatic interactions with ions and PI(4,5)P_2_. The membrane clusters are further refined by protein–protein interactions in the aqueous space.

In summary, we have shown that hydrophobic mismatch due to cholesterol-mediated thickening of the membrane can drive clustering of proteins in distinct membrane domains and that this clustering can be modulated by homotypic interactions of the TMD and electrostatic protein–lipid interactions (Fig. 4). These data provide an alternative to previously established models of plasma membrane patterning^4^,^37^, allowing for drawing of three major conclusions. One, differences between membrane thickness and the length of the hydrophobic TMDs can drive clustering of membrane proteins. The resulting free energy penalty due to hydrophobic mismatch can be described in terms of a line tension, which is minimized by protein–protein clustering. Such hydrophobic mismatch not only explains protein clustering in the plasma membrane, but also may explain cargo sorting of proteins into specific intracellular compartments^26^,^38^,^39^. Indeed, theoretical calculations of hydrophobic mismatch carried out many years ago revealed that ‘embedded inclusions’ (that is, membrane proteins) perturb the membrane thickness, resulting in a state with defined spacing between neighbouring ‘inclusion’ clusters^40^,^41^. Membrane proteins can thus recruit lipids and other membrane proteins with matching hydrophobic thicknesses and are thereby able to pattern biological membranes^42^.

Two, inclusion of physiological concentrations of cholesterol (~30 mol%) results in a substantial thickening (by ~0.8 nm) of the membranes and consequently profoundly increases membrane cluster formation by hydrophobic mismatch. This agrees well with previous studies in which leucine–alanine-rich repeat peptides were used as models for TMDs (refs 24, 25). Our results provide a mechanistic explanation for cholesterol-induced syntaxin 1 domains reported by Murray and Tamm^15^,^16^ and also explain the cholesterol dependence of syntaxin 1 clustering observed in the plasma membrane^5^,^21^. However, in contrast to former interpretations^19^,^43^, we now show that cholesterol-induced cluster formation is the result of hydrophobic mismatch and does not require partitioning into separate membrane phases or domains enriched in cholesterol and sphingolipids (‘lipid rafts’).

Three, clustering of proteins in the plasma membrane can be further modulated by electrostatic interactions with phosphoinositides and ions. Here ions and the charged phosphoinositide head groups can balance the charges of the positive residues found on the juxtamembrane regions of many proteins, thereby overcoming electrostatic repulsion^14^,^32^. We recently showed that polyanionic phosphoinositides can even reinforce the clusters by providing ‘charge bridges’^10^,^18^. At least in the case of syntaxins, homophilic interactions between the TMDs can also promote clustering especially in thinner membranes where the increased rotational mobility facilitates these interactions. Based on our data, we propose a model (Fig. 4) where hydrophobic mismatch and electrostatic protein–protein and protein–lipid interactions act synergistically and are required for the stabilization of syntaxin clusters in the plasma membrane. Finally, homotypic interactions between the SNARE motifs and/or heterotypic interactions with known binding partners (for example, SNAP 25, Munc 18a) can capitalize on the underlying physical principles, increasing specificity of segregation by hydrophobic mismatch due to differences in TMD-length^9^,^22^.

Our findings are important for the understanding of the cellular organization of membrane trafficking. It is well-established that SNARE clusters act as functional platforms for vesicle docking and fusion, either directly 5,44,45,46 or by providing pools of active SNAREs (discussed in ref. 47). Membrane clusters of syntaxin 1 are involved in Ca^2+^-regulated secretion and are completely excluded from clusters of syntaxin 4 involved in constitutive exocytosis^8^,^48^. Our results show that this segregation is at least partially caused by hydrophobic mismatch between the 1–2 residue longer transmembrane helix of syntaxin 4 compared with syntaxin 1. The recruitment of hydrophobically matching lipids and membrane proteins by these SNAREs may also have functional consequences for membrane fusion. It is conceivable that the increased line tension of the membrane clusters induces local membrane curvature and lowers the energy barrier for membrane fusion^49^,^50^,^51^, which could reduce the number of SNARE complexes required for fusion^52^,^53^,^54^. Thus, an important concept raised in this study is that membrane proteins not only seem to partition into lipid-dependent membrane domains, but that they are in fact essential determinants of these lipid domains in the first place. Given that proteins are very abundant in the plasma membrane (20–25 volume% of membrane)^55^,^56^, these protein domains warrant being further investigated as a distinct membrane phase.

## Methods

### Proteins and lipids

Syntaxin 1 TMD (residues 266–288; sx-1TM) from *Rattus norvegicus*, syntaxin 4 TMD (residues 262–297; sx-4TM) from *Homo sapiens* and syntaxin 1 TMD mutant (sx-1TM with the following mutations: M267A, C271A and I279A) were synthesized using Fmoc solid phase synthesis. The fluorescent dyes Atto647N NHS-ester (Atto-Tec) and Rodamine red succinimidyl ester (Life Technologies) were coupled to the N-termini of sx-TM. The detailed synthesis is described in ref. 10.

C18:1 (1,2-dioleoyl-*sn-*glycero-3-phosphocholine), C14:1 (1,2-dimyristoleoyl-*sn-*glycero-3-phosphocholine), C16:1 (1,2-dipalmitoleoyl-*sn-*glycero-3-phosphocholine), C20:1 (1,2-dieicosenoyl-*sn-*glycero-3-phosphocholine), brain PI(4,5)P_2_, doPI(4,5)P_2_ (1,2-dioleoyl-*sn-*glycero-3-phosphatidyl-(1′-myo-inositol-4′,5′-bisphosphate)) and cholesterol were purchased from Avanti Polar Lipids. Bodipy-labelled PI(4,5)P_2_ (bodipy-FL-PI(4,5)P_2_, C16) was from Echelon Biosciences, and Top-Fluor labelled PI(4,5)P_2_ was from Avanti Polar Lipids. The lipophilic fluorescent probe DiO was from Life Technologies.

### Vesicle formation

LUVs were prepared from PC of different acyl-chain lengths (C14:1, C16:1, C18:1 and C20:1) with or without 30 mol% cholesterol and/or 1 mol% PI(4,5)P_2_. Lipid mixtures were prepared at a total concentration of ~30 mM lipids in chloroform as described in ref. 57. After removal of chloroform with a rotary evaporator, the lipid film was resuspended in methanol to the final concentration of 40 mM and fluorescently labelled peptides were added in 2,2,2-trifluoroethanol. The organic solvents were then evaporated and resuspended to 8 mM total lipid concentration in 50 mM HEPES buffer with 150 mM KCl (pH 7.4) unless otherwise indicated (that is, no salt or 1 M NaCl). The multilamellar vesicles (MLVs) were then extruded through polycarbonate filters with 100 nm pore diameter (Avanti Polar Lipids).

### FRET measurements

For FRET analysis we used Rhodamine Red coupled to sx-TM (donor) and Atto647N coupled to sx-TM (acceptor). Protein-to-lipid ratio in our FRET measurements was 1:1000. Excitation was at 560 nm, and emission was collected from 570 to 700 nm with 1 nm slit widths on a FluoroMax-2 (Horiba). We corrected for cross-talk residing from acceptor excitation with samples containing only the acceptor fluorophore. The obtained FRET spectra were normalized to the maximum donor emission at 580 nm. The FRET efficiency was calculated as the ratio of emission intensities at 660 nm (acceptor maximum) over 580 nm (donor maximum)^15^,^16^.

### Protein reconstitutions in polymer-supported membranes

Glass cover slides used in microscopy were prepared as described in ref. 58 and the supported lipid bilayer was generated by spincoating. For spincoating, (at 100 × *g*) we prepared a 40 mM lipid mixture consisting of a 2:1 molar ratio of phospholipids to cholesterol^59^. Molar ratios of DiO and sx-1TM to phospholipids were 1:5,000 and 1:10,000, respectively. After spincoating 10 μl of the lipid solution, the lipid film was rehydrated in 1 ml of 50 mM HEPES buffer with 150 mM KCl (pH at 7.4).

### Cell culture and immunofluorescence

We used the pheochromocytoma cell line PC12 from *R. norvegicus*^60^. Lipofectamin LTX reagents from Life Technologies were used for transfection and cells were analyzed 24 h post transfection. Native membrane sheets were generated by gentle sonication as described in refs 8, 10 and sonication buffer containing 20 mM K-HEPES pH 7.4, 120 mM K-gluconate, 20 mM K-acetate, 2 mM ATP and 0.5 mM DTT. Antibodies used for immunohistochemistry were syntaxin 1 HPC-1 immunoglobulin-G1 (IgG1, Sigma, clone HPC-1) and rabbit polyclonal antiserum (Synaptic Systems); syntaxin 4 mouse monoclonal IgG1 (Synaptic Systems, clone number 139.2); IgM antibodies against PI(4,5)P_2_ (clone Z-A045); mouse monoclonal IgG2a anti-mCherry (Abcam, clone 1C51) and rabbit polyclonal anti-EGFP (Abcam). Secondary antibodies against IgG and IgM were labelled with Alexa Fluor 488 C_5_-maleimide (Life Technologies) or KK114-maleimide (gift from Vladimir Belov, MPI-BPC, Göttingen, Germany, as described in ref. 61).

For transfection of PC12 cells, we used synthetic chimeric constructs (Genscript) in the KpnI–HindIII restriction sites of pCEP4. The sequences for syntaxin 1A (sequence from *R. norvegicus* 262–297) N-terminally tagged with mCherry or mEGFP are given in Supplementary Table 1. The constructs coding for mCherry-tagged sx-1TM^−IFG^ (residues 257–285) and sx-1TM^+VG^ (residues 257–288 with two additional amino acids at the C terminus: V289, G290) were generated from the wild-type construct by Quick Change mutagenesis (Agilent Technologies).

### Fluorescence correlation spectroscopy

For FCS, we used home-built confocal beam-scanning microscopy set up with two-colour excitation by pulsed-diode lasers at 485 nm (pulse length 80 ps; LDH-P-635, PicoQuant) and 635 nm (pulse length 80 ps LDH-P-485B, PicoQuant)^62^. Emission filters were 540±20 for the green channel and 670±30 for the red channel. We used a × 100 oil objective with 1.42 numerical aperture (Leica Microsystems). Avalanche single photon counting detectors were used (SPCM-AQR-13-FC, Perkin Elmer Optoelectronics). FCS curves were fitted to a model for two-dimensional diffusion and with the axial radii of the focal volumes *ω*_*xy*,_ defined as the point where the measured fluorescence drops *e*^2^ times relative to the maximum, of 200 and 250 nm for Bodipy-FL and Atto647N, respectively.

### Two-colour STED microscopy

The STED images were acquired on a home-built set up as described in ref. 36. The basic set up resembles a standard confocal microscope with pulsed excitation at 595 nm and 640 nm wavelength. The fluorescence was collected from 600–640 nm and 660–720 nm by avalanche photo diodes (Excelitas, USA and Micro Photon Devices, Italy), allowing good spectral separation of the dyes used. Super-resolution was achieved by silencing the fluorophores in the periphery of the diffraction-limited excitation spot via stimulated emission induced by the STED laser, a 775 nm wavelength, 20 MHz pulsed fibre laser (IPG Photonics). In combination with a 2π vortex phase plate (RPC Photonics, USA) and a *λ*/4 plate the typical ‘doughnut’-shaped focal intensity distribution with its central zero was produced. Pulse energies from 3 to 8 nJ in the objectives back aperture yield a resolution of down to 30 nm. Using the same STED beam for both dyes inherently ensures a co-localization accuracy far below the resolution limit. As we record both colour channels quasi simultaneously, we do not have to correct for drift or channel misalignment. The hardware and data acquisition was controlled by the software ImSpector ( http://www.imspector.de). The density of clusters was analyzed using the particle analysis plugin in the Fiji software^63^.

### Ellipsometry measurements

Lipid stock solutions (*c*_lipid_=1–10 mg ml^−1^) were prepared in chloroform and transformed into lipid films by removal of the solvent in a nitrogen stream followed by 3 h drying in vacuum. MLV were produced by resuspending the lipid films in buffer (50 mM HEPES pH 7.4 with 3 mM Ca^2+^) at a concentration of 1 mg ml^−1^. MLVs were transformed into small unilamellar vesicles by sonication (50 W, 0.4 s pulse, 30 min) in a vessel resonator (Sonoplus HD 2070). Average vesicle size was 30–50 nm as determined by dynamic light scattering^64^. Si-Wafers were cleaned in H_2_O_2_/NH_3_/H_2_O 1:1:5 at 70 °C for 15 min and afterwards hydrophilized for 1 min in O_2_ plasma. For preparing the lipid bilayer, freshly prepared small unilamellar vesicles were spread for 10–30 min on a hydrophilized Si-Wafer at a concentration of 0.2 mg ml^−1^ in 50 mM HEPES pH 7.4 with 3 mM Ca^2+^. Measurements were carried out in the same buffer in a closed fluid chamber. Ellipsometry experiments were performed using an imaging ellipsometer EP^3^-SW from Accurion as described previously^29^,^65^. This method offers the possibility to measure thin layer thicknesses in real time within a convective flow at defined temperature. The principal angle del determined by this method is proportional to layer thicknesses for sufficiently thin dielectric layers (*h*<30 nm). Absolute height changes resulting from spread solid-supported membranes were computed from the angle del, which is linearly related to the height for thin layers (1 nm≈0.91° del) and assuming a refractive index of 1.5 for all the used lipids.

### Molecular dynamics

The molecular dynamics simulations were performed with the GROMACS simulation package, version 4.5.5 ref. 66. We used the MARTINI coarse-grained model to simulate the lipids, amino acids and polar solvents^67^,^68^. Within this model an unsaturated bond is modelled by a decreased equilibrium bond-angle (120° versus 180°), increased flexibility and increased polarity. In all simulations, the system was coupled to a constant temperature bath with a relaxation time of 1.0 ps ref. 69. We performed our simulations at a temperature of 310 K. Periodic boundary conditions where applied to simulate bulk behaviour. The time step used in the simulation was 20 fs. The dielectric constant in the simulations was *ε*_r_=15. The neighbor list was updated every 10 simulation steps. The pressure was weakly coupled to 1 bar with a relaxation time of 0.5 ps. In analogy to the other studies done with the MARTINI model, the time scales quoted in this work were scaled by a factor of 4 to approximately correct for the faster diffusion rates of water and lipids in the coarse-grained model^48^.

Sx-1TM (residues 266–288) was modelled using the MARTINI model for proteins, which qualitatively captures the chemical nature of each individual amino acid and includes the secondary structure. In our simulations, sx-1TM and linker region (residues 257–265) were modelled as an α-helix. The backbone was modelled as a stiff and conserved spring with a length of 3.6 nm (residue 266–288). This model allows us to qualitatively investigate the relationship between membrane thickness, free energy of protein insertion (hydrophobic mismatch) and protein clustering.

The simulations used to study spontaneous sx-1TM clustering contained 81 copies of sx-1TM embedded in a 30 × 30 μm planar bilayer containing 2,560 lipids and 43,000 solvent beads. The total charge of the system was kept at zero with Cl^−^ counterions. To mimic the cell membrane, all sx-1TM peptides were embedded with their N-termini facing the same side of the membrane. These simulations were run for 60 μs. Additionally, we also performed 4 μs simulations with a smaller 7 × 7 nm bilayer system (128 lipids) that contained a single sx-1TM. These latter simulations were used to calculate the membrane thickness adaptation and membrane insertion free energy of sx-1TM. The cluster-sizes (oligomer size) were calculated over the last 20 μs, using a distance cutoff-based cluster algorithm. Neighbouring sx-1TMs were considered clustered if any of the beads in the transmembrane region (residues 266–288) were within a distance of 0.7 nm.

To provide a quantitative measure for the hydrophobic mismatch, that is, the protein–lipid interactions, we applied thermodynamic integration techniques (with 4 μs simulation for each lambda point) to calculate the relative free energy of membrane insertion for sx-1TM as a function of membrane thickness/composition. The exact protocol can be found elsewhere^25^.

## Additional information

**How to cite this article:** Milovanovic, D. *et al.* Hydrophobic mismatch sorts SNARE proteins into distinct membrane domains. *Nat. Commun.* 6:5984 doi: 10.1038/ncomms6984 (2015).

## Supplementary Material

Supplementary Information Supplementary Figures 1-2, Supplementary Table 1 and Supplementary Reference.
