# Supplementary material for: Hydrophobic mismatch sorts SNARE proteins into distinct membrane domains
Source: Nat Commun. 2015 Jan 30;6:5984. doi: 10.1038/ncomms6984 (PMC4313621; doi:10.1038/ncomms6984)
Supplement: Supplementary Information — Supplementary Figures 1-2, Supplementary Table 1 and Supplementary Reference. [file ncomms6984-s1.pdf]

Supplementary Figures

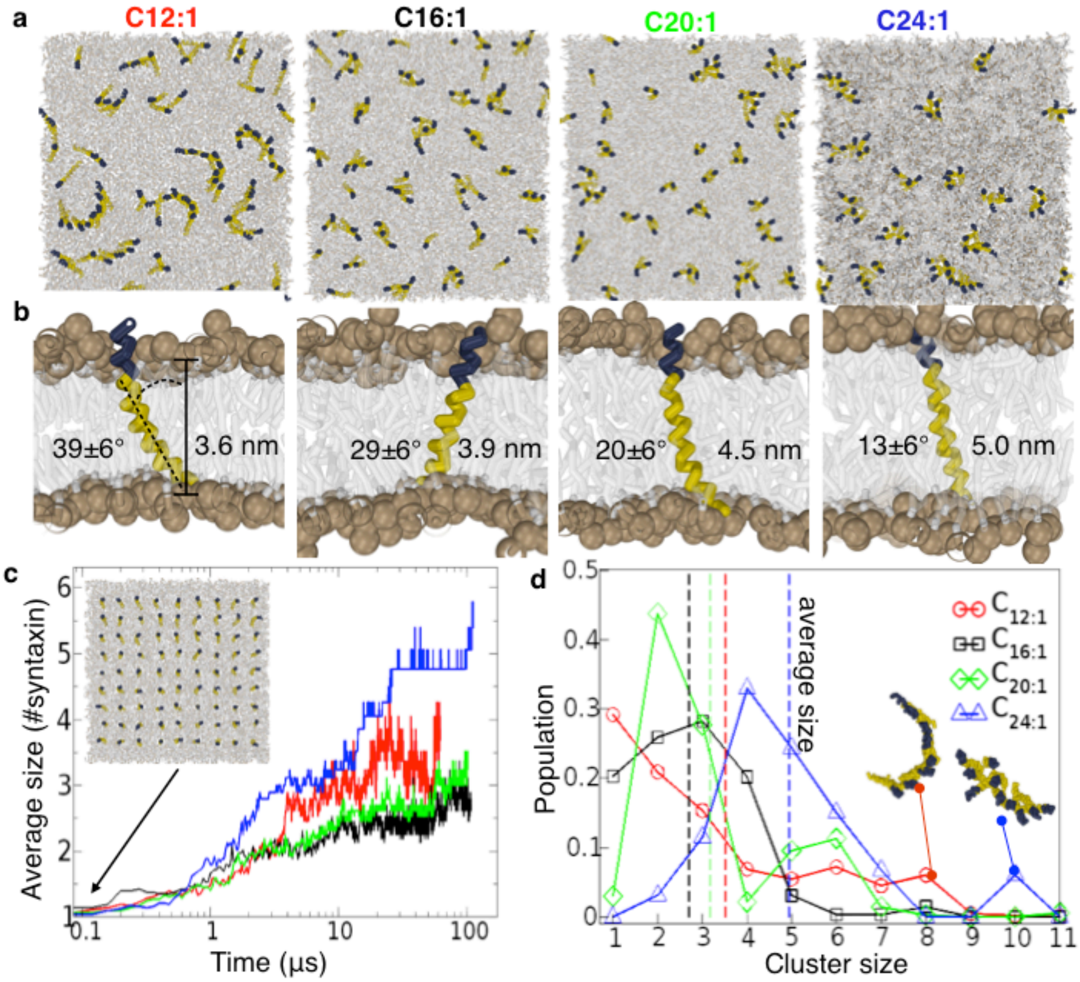

Supplementary figure 1 | Molecular dynamics simulations. (**a**) Snapshots after 100 μs simulation run in membranes of C12:1, C16:1, C20:1 and C24:1 PC. (**b**) Side view of a single sx-1TM in the corresponding membrane with the hydrophobic thicknesses and the average tilt angles indicated (relative to the normal of the membrane, +/- standard deviation) indicated. (**c**) Time evolution of the average cluster growth during the simulation runs. (**d**) Distributions of average cluster sizes from the simulation runs indicate that sx-1TM formed clusters of typically less than 10 peptide units. Note the highly regular, crystalline shape of the larger oligomers formed in the C12:1 PC membrane (further characterized in Supplementary Fig. 2c-e). The shape of the distribution in C12:1 PC, i.e. monomers being the most abundant with each successive

larger species becoming increasingly more rare, suggests that the growth of clusters proceeds via an alternative nucleated mechanism.

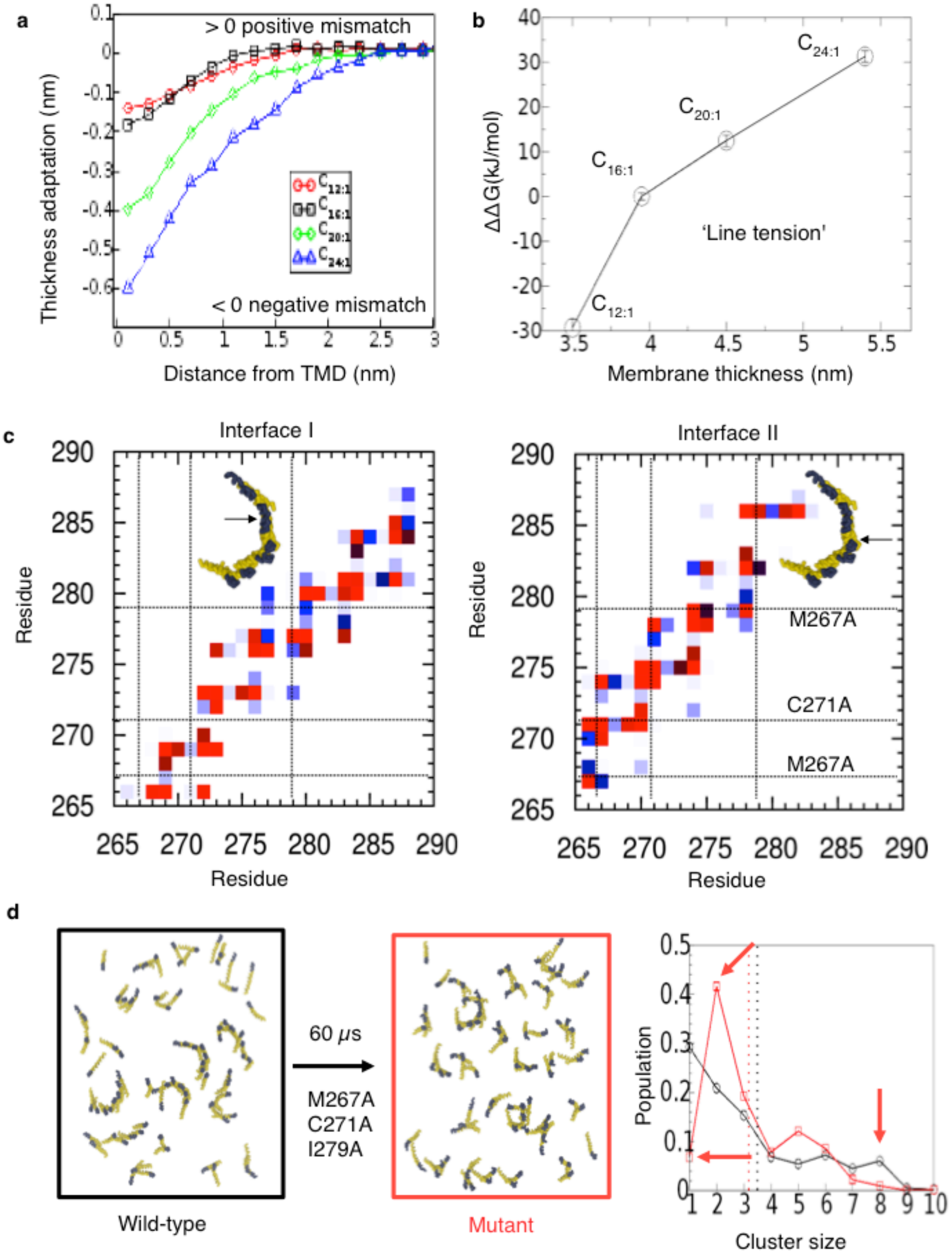

Supplementary figure 2 | Hydrophobic mismatch and protein-protein interactions promote clustering of sx-1TM. (**a**) Adaption of membrane thickness due to hydrophobic

mismatch in the presence of a single sx-1TM in the 7 x 7 nm bilayer with 128 lipids. (**b**) The hydrophobic mismatch changes with membrane thickness and is quantified by the relative insertion free energy ΔΔG (or 'line tension', when normalized by the TMD extension of the hydrophobic mismatch interface) of sx-1TM in the membrane of the systems described in **a**. All values provided are relative with respect to the insertion free-energy of C16:1. These free-energies suggest that the peptides most favorably partition in C12:1 membranes, indicating that the increased clustering in C12:1 is not driven by lipid-protein interactions. (**c**) Example of the two different TMD-TMD interfaces formed in an octamer in the C12:1 membrane. TMD-TMD interfaces are known to be membrane thickness dependent (e.g., differences in orientation), and can outperform an unfavorable increase in hydrophobic mismatch[1]. The color code (from white to red) indicates the frequency at which contacts between the residues are formed. The mutations M267A, C271A, I279A (indicated with dotted black lines) mainly affect the interactions in the second interface, while leaving the first interface intact. (**d**) Effect of the mutations on the formed clusters in the C12:1 membrane. The mutations destabilize the larger oligomers and markedly affect the shape of the wild-type size distribution. Interestingly, this modified size distribution in the C12:1 membrane closely resembles the wild type size distribution (Supplementary Fig. 1d) seen for mainly line tension driven clustering in membranes with longer acyl chains.

Supplementary Tables

Supplemetary table 1 | Syntaxin 1A and syntaxin 4 TMD sequences used for PC12 cell transfections.

**1. Syntaxin 1A (sequence from *Rattus norvegicus*, residues 257-288) N-terminally tagged with mCherry**
5’ATGGTGAGCAAGGGCGAGGAGGACAACATGGCCATCATCAAGGAGTTCAT<br>GAGGTTCAAGGTGCACATGGAGGGCAGCGTGAACGGCCACGAGTTCGAGAT<br>CGAGGGCGAGGGCGAGGGCAGGCCCTACGAGGGCACCCAGACCGCCAAGCT<br>GAAGGTGACCAAGGGCGGCCCCCTGCCCTTCGCCTGGGACATCCTGAGCCCC<br>CAGTTCATGTACGGCAGCAAGGCCTACGTGAAGCACCCCGCCGACATCCCCG<br>ACTACCTGAAGCTGAGCTTCCCCGAGGGCTTCAAGTGGGAGAGGGTGATGAA<br>CTTCGAGGACGGCGGCGTGGTGACCGTGACCCAGGACAGCAGCCTGCAGGA<br>CGGCGAGTTCATCTACAAGGTGAAGCTGAGGGGCACCAACTTCCCCAGCGAC<br>GGCCCCGTGATGCAGAAGAAGACCATGGGCTGGGAGGCCAGCAGCGAGAGG<br>ATGTACCCCGAGGACGGCGCCCTGAAGGGCGAGATCAAGCAGAGGCTGAAG<br>CTGAAGGACGGCGGCCACTACGACGCCGAGGTGAAGACCACCTACAAGGCC<br>AAGAAGCCCGTGCAGCTGCCCGGCGCCTACAACGTGAACATCAAGCTGGACA<br>TCACCAGCCACAACGAGGACTACACCATCGTGGAGCAGTACGAGAGGGCCG<br>AGGGCAGGCACAGCACCGGCGGCATGGACGAGCTGTACAAGGGCGGCCACA<br>GGTGGATCTACCAGAGCAAGGCCAGGAGGAAGAAGATCATGATCATCATCT<br>GCTGCGTGATCCTGGGCATCGTGATCGCCAGCACCGTGGGCGGCATCTTCGC<br>C3’
**2. Syntaxin 1A (sequence from *Rattus norvegicus*, residues 257-288) N-terminally tagged with mEGFP**
5’GGTACCATGGTGAGCAAGGGCGAGGAGCTGTTCACCGGCGTGGTGCCCATC<br>CTGGTGGAGCTGGACGGCGACGTGAACGGCCACAAGTTCAGCGTGAGCGGC<br>GAGGGCGAGGGCGACGCCACCTACGGCAAGCTGACCCTGAAGTTCATCTGCA<br>CCACCGGCAAGCTGCCCGTGCCCTGGCCCACCCTGGTGACCACCCTGACCTA<br>CGGCGTGCAGTGCTTCAGCAGGTATCCCGACCACATGAAGCAGCACGACTTC<br>TTCAAGAGCGCCATGCCCGAGGGCTACGTGCAGGAGAGGACCATCTTCTTCA<br>AGGACGACGGCAACTACAAGACCAGGGCCGAGGTGAAGTTCGAGGGCGACA<br>CCCTGGTGAACAGGATCGAGCTGAAGGGCATCGACTTCAAGGAGGACGGCA<br>ACATCCTGGGCCACAAGCTGGAGTACAACTACAACAGCCACAACGTGTACAT<br>CATGGCCGACAAGCAGAAGAACGGCATCAAGGTGAACTTCAAGATCAGGCA<br>CAACATCGAGGACGGCAGCGTGCAGCTGGCCGACCACTACCAGCAGAACAC<br>CCCCATCGGCGACGGCCCCGTGCTGCTGCCCGACAACCACTACCTGAGCACC<br>CAGAGCAAGCTGAGCAAGGACCCCAACGAGAAGAGGGACCACATGGTGCTG<br>CTGGAGTTCGTGACCGCCGCCGGCATCACCCTGGGCATGGACGAGCTGTACA<br>AGGGCGGCCACAGGTGGATCTACCAGAGCAAGGCCAGGAGGAAGAAGATCA<br>TGATCATCATCTGCTGCGTGATCCTGGGCATCATCATCGCCAGCACCATCGGC<br>GGCATCTTCGGCTAGTAAAAGCTT3’
**3. Syntaxin 4 (sequence from *Rattus norvegicus*, residues 262-297) N-terminally tagged with mEGFP.**

5’ATGGTGAGCAAAGGCGAAGAACTGTTTACCGGCGTGGTGCCGATTCTGGTG
GAACTGGATGGCGATGTGAACGGCCATAAATTTAGCGTGAGCGGCGAAGGC
GAAGGCGATGCGACCTATGGCAAACTGACCCTGAAATTTATTTGCACCACCG
GCAAACTGCCGGTGCCGTGGCCGACCCTGGTGACCACCCTGACCTATGGCGT
GCAGTGCTTTAGCCGCTATCCGGATCATATGAAACAGCATGATTTTTTAAAA
GCGCGATGCCGGAAGGCTATGTGCAGGAACGCACCATTTTTTTTAAAGATGA
TGGCAACTATAAAACCCGCGCGGAAGTGAAATTTGAAGGCGATACCCTGGTG
AACCGCATTGAACTGAAAGGCATTGATTTTAAAGAAGATGGCAACATTCTGG
GCCATAAACTGGAATATAACTATAACAGCCATAACGTGTATATTATGGCGGA
TAAACAGAAAAACGGCATTAAAGTGAACTTTAAAATTCGCCATAACATTGAA
GATGGCAGCGTGCAGCTGGCGGATCATTATCAGCAGAACACCCCGATTGGCG
ATGGCCCGGTGCTGCTGCCGGATAACCATTATCTGAGCACCCAGAGCAAACT
GAGCAAAGATCCGAACGAAAAACGCGATCATATGGTGCTGCTGGAATTTGTG
ACCGCGGCGGGCATTACCCTGGGCATGGATGAACTGTATAAAGGCGGCCATC
GCTGGATTGCGCTGGAAAACCAGAAAAAAGCGCGCAAAAAAAAAGTGCTGA
TTGCGATTTGCGTGAGCATTACCGTGGTGCTGCTGGCGGTGATTATTGGCGTG
ACCGTGGTGGGC3’

Supplementary Reference

1. Flinner, N., Mirus, O., Schleiff, E. The influence of fatty acids on the GpA dimer interface by coarse-grained molecular dynamics simulation. *Int. J. Mol. Sci.* **15,** 14247–14268 (2014).
